# The Contribution of ArsB to Arsenic Resistance in *Campylobacter jejuni*

**DOI:** 10.1371/journal.pone.0058894

**Published:** 2013-03-15

**Authors:** Zhangqi Shen, Jing Han, Yang Wang, Orhan Sahin, Qijing Zhang

**Affiliations:** 1 Department of Veterinary Microbiology and Preventive Medicine, Iowa State University, Ames, Iowa, United States of America; 2 Department of Pharmacology and Toxicology, College of Veterinary Medicine, China Agricultural University, Beijing, China; Cornell University, United States of America

## Abstract

Arsenic, a toxic metalloid, exists in the natural environment and its organic form is approved for use as a feed additive for animal production. As a major foodborne pathogen of animal origin, *Campylobacter* is exposed to arsenic selection pressure in the food animal production environments. Previous studies showed that *Campylobacter* isolates from poultry were highly resistant to arsenic compounds and a 4-gene operon (containing *arsP*, *arsR*, *arsC*, and *acr3*) was associated with arsenic resistance in *Campylobacter*. However, this 4-gene operon is only present in some *Campylobacter* isolates and other arsenic resistance mechanisms in *C. jejuni* have not been characterized. In this study, we determined the role of several putative arsenic resistance genes including *arsB*, *arsC2*, and *arsR3* in arsenic resistance in *C. jejuni* and found that *arsB*, but not the other two genes, contributes to the resistance to arsenite and arsenate. Inactivation of *arsB* in *C. jejuni* resulted in 8- and 4-fold reduction in the MICs of arsenite and arsenate, respectively, and complementation of the *arsB* mutant restored the MIC of arsenite. Additionally, overexpression of *arsB* in *C. jejuni* 11168 resulted in a 16-fold increase in the MIC of arsenite. PCR analysis of *C. jejuni* isolates from different animals hosts indicated that *arsB* and *acr3* (the 4-gene operon) are widely distributed in various *C. jejuni* strains, suggesting that *Campylobacter* requires at least one of the two genes for adaptation to arsenic-containing environments. These results identify ArsB as an alternative mechanism for arsenic resistance in *C. jejuni* and provide new insights into the adaptive mechanisms of *Campylobacter* in animal food production environments.

## Introduction

Arsenic is a wildly distributed toxic metalloid in water, soil, and air from natural and anthropogenic sources, and exists in both inorganic and organic forms [1]–[3]. The most prevalent inorganic forms of arsenic include trivalent arsenite [AS(III)] and pentavalent arsenate [AS(V)]. The trivalent form is more toxic than the pentavalent form [1], [3]. AS(III) impairs the functions of many proteins by reacting with their sulfhydryl groups, while AS(V) is a molecular analog of phosphate, which inhibits oxidative phosphorylation and harms the main energy-generation system [2], [4]. In order to survive arsenic toxicity, microorganisms have developed different mechanisms for arsenic detoxification, including reduction of AS(V) to AS(III) by arsenate reductases and methylation or extrusion of AS(III) by efflux transporters [5]–[7].

The genes encoding arsenic detoxification systems are found on both plasmids and chromosomes. Usually, the *ars* genes are organized as operons, such as *arsRBC*, *arsRABC*, and *arsRDABC*, but some *ars* genes exist singly [6], [8]–[14]. ArsC is a small-molecular mass arsenate reductase, which converts AS(V) to AS(III) in the cytoplasm [5], [15]. As(III) is extruded by AS(III)-specific transporters, such as ArsB and Acr3 [5], [6]. The activity of ArsB can be ATP-independent or requires the help of ArsA, an ATPase [16], [17]. A recent study identified a new arsenic detoxification mechanism mediated by ArsM, an AS(III) S-adenosylmethionine methyltransferase, which methylates AS(III) to volatile trimethylarsine [7]. In addition, there are other Ars proteins involved in arsenic resistance. ArsR, a transcription regulator, modulates the expression of arsenic resistance genes [6], [18]–[21]. ArsD, an arsenic metallochaperone, transfers As(III) to ArsA and increases the rate of arsenic extrusion [7], [22]–[24]. ArsH, an NADPH-flavin mononucleotide oxidoreductase, also contributes to arsenic resistance, and its detoxification mechanism is probably through oxidation of arsenite to the less toxic arsenate or reduction of trivalent arsenicals to volatile arsines that escape from cells [25], [26].

*Campylobacter* is a leading cause of food-borne bacterial diseases in the United States and other developed countries [27]. *Campylobacter* infections account for 400 to 500 million cases of diarrhea each year worldwide [28]. According to a recent CDC report, campylobacteriosis is estimated to affect over 840,000 people every year in the U. S. [27]. As a zoonotic pathogen, *Campylobacter* is highly prevalent in food producing animals, including both livestock and poultry [29], and is frequently exposed to antimicrobials used in animal agriculture. Roxarsone (4-hydroxy-3-nitro-phenylarsonic acid), an organoarsenic compound, is frequently used as a feed additive to improve weight gain, feed utilization and pigmentation, and control of coccidiosis in the poultry industry [30]. Organic roxarsone is excreted through feces and can also be converted into inorganic AS(V) and AS(III) in the broiler digestive system, and the total arsenic concentration in the litter can reach up to 39 mg/kg [31], [32]. Due to the concern with food safety, the manufacturer of roxarsone voluntarily suspended sale of this product in the U.S. in 2011 (http://www.fda.gov/AnimalVeterinary/SafetyHealth/ProductSafetyInformation/ucm258313.htm). Given that *Campylobacter* is prevalent and well adapted in poultry digestive system, this organism must have the ability to deal with the toxicity of arsenic compounds used for poultry production.

Recently, Wang *et al*. identified a 4-gene *ars* operon, which is associated with high-level arsenic resistance in *Campylobacter* [6]. This operon encodes a putative membrane permease (ArsP), a transcriptional repressor (ArsR), an arsenate reductase (ArsC), and an efflux protein (Acr3). The expression of the whole operon is directly regulated by ArsR and is inducible by AS(III) and As(V) [6]. According to the published whole genome sequences of *Campylobacter*, this *ars* operon is not present in all *Campylobacter* strains and how those strains without this *ars* operon adapt to arsenic selection is unknown. The first sequenced *C. jejuni* strain NCTC 11168 (http://www.lshtm.ac.uk/pmbu/crf/Cj_updated.art) lacks the previously characterized *ars* operon [6], [33], but three putative *ars* genes are present on the chromosome. These include *cj0258* (an *arsR* homolog and named *arsR3* in this study), *cj0717* (an *arsC* homolog and named *arsC2* in this study), and *cj1187c* (an *arsB* homolog and named *arsB* in this study), and their functions remain unknown. In this study, we determine the roles of these putative *ars* genes in arsenic resistance and found that *cj1187c* (*arsB*) contributes to the resistance to AS(III). In addition, we investigated the presence of the *arsB* and *acr3* (present in the 4-gene *ars* operon) genes in various *Campylobacter* isolates. The results suggest that *Campylobacter* requires at least one of the two genes for adaptation to arsenic-containing environment.

## Materials and Methods

### Bacterial Strains and Growth Conditions

The key bacterial strains and plasmids used in this study are listed in Table 1. *Escherchia coli* DH5α used for genetic manipulation was grown in Luria-Bertani (LB) broth or on Mueller-Hinton (MH) agar. When required for cloning of plasmids with different selection markers, kanamycin (30 µg/ml), chloramphenicol (10 µg/ml), or ampicillin (100 µg/ml) was added to the culture media. *C. jejuni* strains were cultured on MH agar or in MH broth at 42°C microaerobically (5% O_2_, 10% CO_2_, and 85% N_2_). Kanamycin (30 µg/ml) or chloramphenicol (4 µg/ml) was supplemented to the media when needed for culturing the mutant strains that contained a selection marker.

**Table 1:** Bacterial strains and plasmids used in this study.

Bacterial strain or plasmid	Description or relevant genotype	Source or reference
**Plasmids**		
pUOA18	*E. coli*-*C. jejuni* shuttle vector	[48]
pUC19	Cloning vector	[49]
pMW10	Promoterless *lacZ* plasmid	[50]
pArsB	pUC19*+arsB*	This study
pArsBcat	pUC19*+arsB*::*cat*	This study
pRRK	pRR::*aphA3*	[36]
pRRK*arsB*	pRRK*+arsB*	This study
pC2M1	pUC19*+arsC2M1*	This study
pC2M1M2	pUC19*+arsC2M1+arsC2M2*	This study
pC2M1M2Kan	pUC19*+arsC2M1+aphA3+arsC2M2*	This study
pR2M1	pUC19*+arsR3M1*	This study
pR2M1M2	pUC19*+arsR3M1+arsR3M2*	This study
pR2M1M2Kan	pUC19*+arsR3M1+aphA3+arsR3M2*	This study
**strains**		
DH5α	Plasmid propagation *E.coli* strain	Invitrogen
NCTC 11168	Wild-type *C. jejuni*	[33]
11168Δ*arsB*	NCTC 11168 derivative, Δ*arsB::Cm^r^*	This study
11168Δ*arsC2*	NCTC 11168 derivative, Δ*arsC2:: aphA3*	This study
11168Δ*arsR3*	NCTC 11168 derivative, Δ*arsR3:: aphA3*	This study
11168Δ*arsB*Δ*arsC2*	NCTC 11168 derivative, Δ*arsB::Cm^r^*, Δ*arsC:: aphA3*	This study
11168*+arsB*	NCTC 11168 derivative, *rrs::arsB*	This study
11168Δ*arsB+arsB*	11168ΔarsB derivative, *rrs::arsB*	This study
ATCC 33560	Wild-type *C. jejuni*	ATCC
33560Δ*arsB*	ATCC 33560 derivative, Δ*arsB::Cm^r^*	This study
CB5-28	Wild-type *C. jejuni*	[6]
CB5-28Δ*arsB*	CB5-28 derivative, Δ*arsB::Cm^r^*	This study
CB5-28Δ*arsC*	CB5-28 derivative, Δ*arsC:: aphA3*	[6]
CB5-28Δ*arsB*Δ*arsC*	CB5-28 derivative, Δ*arsB::Cm^r^* Δ*arsC:: aphA3*	This study

### Chemical Compounds and Antibiotics

The chemicals and antibiotics used in this study were purchased from Sigma-Aldrich Co. LLC (arsenite, arsenate, chloramphenicol, kanamycin, ampicillin, copper sulfate, erythromycin, tetracycline, ethidium bromide, azithromycin, ciprofloxacin, florfenicol, and clindamycin), Thermo Fisher Scientific Inc. (roxarsone, mercury bichloride, and telithromycin), and Alfa Aesar (antimonite).

### Antimicrobial Susceptibility Tests

The MICs of various arsenic compounds against *C. jejuni* strains were determined using the agar dilution antimicrobial susceptibility testing method according to the protocol from CLSI [34]. The concentrations of arsenic compounds tested in this study ranged from 0.25 to 256 µg/ml for arsenite, 2 to 2048 µg/ml for arsenate, and 1 to 512 µg/ml for roxarsone. Brieﬂy, *Campylobacter* strains grown on blood agar plates for 24 h were inoculated into Mueller-Hinton broth and then adjusted to a turbidity equivalent to a 0.5 McFarland standard by a colorimeter. A multipoint inoculators (a Cathra replicator system) with 1-mm pins (Oxoid, Inc., Ogdensburg, NY) was used to inoculate approximately 10^4 ^CFU of *C. jejuni* onto Mueller-Hinton agar containing a twofold dilution series of arsenic compounds and supplemented with 5% defibrinated sheep blood. The inoculated plates were incubated at 42°C microaerobically (5% O_2_, 10% CO_2_, and 85% N_2_). The MIC was defined as the lowest concentration that completely inhibited the visible growth on the plates. The MICs of various antibiotics against *C. jejuni* strains were determined using the broth microdilution method as described previously [35]. Each MIC test was repeated at least three times.

### PCR

All primers used for PCR are listed in Table 2. PCR was performed in a volume of 50 µl containing 0.2 µM of primers, 250 µM of deoxynucleoside triphosphates, and 1.25 U of TaKaRa Ex Taq polymerase or Phusion High-Fidelity DNA Polymerase. The annealing temperature varied from 50°C to 58°C (Table 2) and the elongation time dependent on the expected size of the products (1 kb/min).

**Table 2:** PCR primers used in this study.

Primers	Sequence (5′→3′)	Annealing temperature (°C)
arsB1929F	ACAAGGAATTCATGGCTATGATTTAGGGC	56
arsB1929R	ATCATGTCGACCCATAACTTGTCCTTTCG	56
arsBCat-F	CGGTTCTAGATGGAGCGGACAACGAGTAAA	58
arsBCat-R	GCTTGGATCAGTGCGACAAACTGGGATT	58
comarsB-F	GCCGCTAGCAAGGAGATTTAAATGCTTGCTTTTTTTATTTTTTT	52
comarsB-R	GGTGCTAGCTTAGACAATAAGAGCAAAAAGAGAA	52
gidAKanF	TATGGTACCCGCTTATCAATATATCTATAGAATG	50
gidAKanR	AGCTCTAGAGATAATGCTAAGACAATCACTAAA	50
arsBgidAF	CATCATAAACCTCCAACCATT	58
arsBgidAR	AAGAACTATCCCAAACCAAG	58
arsR3M1-F	TGGGAATTCGAGGCTTTAATCAACACTTA	52
arsR3M1-R	TAAGGTACCTTTCATCGGCATTTTCACAT	52
arsR3M2-F	CGATCTAGATGTGAAAATGCCGATGAAAA	52
arsR3M2-R	ATTCTGCAGACCATGCACTAGCAAAGGAA	52
arsC2M1-F	TGGGAATTCTTACGATTGTTCAGCTCACA	52
arsC2M1-R	GCTGGTACCAAGCATCCATAGCTTTCTTT	52
arsC2M2-F	TTGTCTAGACCAAGTTGTATTAAGCGTCCT	52
arsC2M2-R	AATCTGCAGCCATGATCTGTCATAGCCAC	52
16sarsB-F	ATCGTAGATCAGCCATGCTA	54
16sarsB-R	GATAATCAACCCAACCAAAGT	54
arsB-F1	AGGATAATCAACCCAACCAAAGT	58
arsB-R1	CGTCCATGGAATTTACCTATTTG	58
arsB56F	GGAATTTACCTATTTGGGTAT	50
arsB1185R	ATATTAATGCCTTTTCTAGCC	50
cje1733F	ATGTTAGGTTTTATCGATAGAT	50
cje1733R	TCATGAGGCTTGATTCATTTTT	50

### Insertional Mutation of *arsB*

Primers arsB1929F and arsB1929R (Table 2) were used to amplify a 1929 bp *arsB* fragment with the *Swa*I and *Xba*I restriction sites in the middle region of the fragment. The PCR fragment was cloned into the pUC19 between the *EcoR*I and *Sal*I sites, resulting in the construction of pArsB. Primers arsBCat-F and arsBCat-R (Table 2) were used to amplify the chloramphenicol resistance *cat* gene from pUOA18 using the Phusion High-Fidelity DNA Polymerase (NEB). After the *Xba*I digestion, the *cat* cassette was ligated to the *Swa*I and *Xba*I digested pARSB to obtain plasmid pArsBcat, which was then transformed into *E. coli* DH5α. Suicide vector pArsBcat was introduced into *C. jejuni* NCTC 11168 using an electroporator (Gene Pulser Xcell System; Bio-Rad Laboratories). Transformants were selected on MH agar containing chloramphenicol at 4 µg/ml. The insertion of *cat* cassette into the *arsB* gene of *C. jejuni* 11168 was confirmed by PCR analysis using primers arsB1929F and arsB1929R.

### Insertional Mutagenesis of *arsC2 (cj0717)*

Primers arsC2M1-F and arsC2M1-R were used to amplify the 5′ part of *arsC2* and its upstream region (arsC2M1), while Primers arsC2M2-F and arsC2M2-R were used to amplify the 3′ part of *arsC2* and its downstream region (arsC2M2). After *EcoR*I and *Kpn*I digestion, the arsC2M1 PCR product was cloned into the *EcoR*I and *Kpn*I digested pUC19, resulting in the construction of pC2M1. The digested arsC2M2 PCR product was cloned into the *Xba*I and *Pst*I digested pC2M1, resulting in the construction of pC2M1M2. Primers gidAKanF and gidAKanR (Table 2) were used to amplify the *aphA3* gene encoding kanamycin resistance from pMW10 using the Phusion High-Fidelity DNA Polymerase (NEB). After the *Kpn*I and *Xba*I digestion, the Kan^r^ cassette was ligated to the *Kpn*I and *Xba*I digested pC2M1M2 to obtain plasmid construct pC2M1M2Kan, which was then transformed into *E. coli* DH5α. Suicide vector pC2M1M2Kan was then electroporated into *C. jejuni* NCTC 11168. Transformants were selected on MH agar plates containing 30 µg/ml of kanamycin. The insertion of the *aphA3* gene into *arsC2* in the transformants was confirmed by PCR using primers arsC2M1-F and arsC2M2-R.

### Insertional Mutagenesis of *arsR3 (cj0258)*

Primers arsR3M1-F and arsR3M1-R were used to amplify the 5′ part of *arsR3* and its upstream region (arsR3M1), while primers arsR3M2-F and arsR3M2-R were used to amplify the 3′ part of *arsR3* and its downstream region (arsR3M2). After *EcoR*I and *Kpn*I digestion, the arsR3M1 PCR product was cloned into the *EcoR*I and *Kpn*I digested pUC19, resulting in the construction of pR3M1. The digested arsR3M2 PCR product was cloned into the *Xba*I and *Pst*I digested pR3M1, resulting in the construction of pR3M1M2. As mentioned above, primers gidAKanF and gidAKanR (Table 2) were used to amplify the *aphA3* gene encoding kanamycin resistance from pMW10 using the Phusion High-Fidelity DNA Polymerase (NEB). After the *Kpn*I and *Xba*I digestion, the Kan^r^ cassette was ligated to the *Kpn*I and *Xba*I digested pR3M1M2 to obtain plasmid construct pR3M1M2Kan, which was then transformed into *E. coli* DH5α. Suicide vector pR3M1M2Kan was then electroporated into *C. jejuni* NCTC 11168. Transformants were selected on MH agar plates containing 30 µg/ml of kanamycin. The insertion of the *aphA3* gene into *arsR3* in the transformants was confirmed by PCR using primers arsR3M1-F and arsR3M2-R.

### Complementation of the Δ*arsB*::*Cm^r^* Mutant

The Δ*arsB*::*Cm^r^* mutant was complemented by inserting a wild-type copy of *arsB* between the 16S and 23S rRNAs as described by Muraoka and Zhang [36]. Briefly, primers comarsB-F and comarsB-R were used to amplify the intact *arsB* gene including its ribosome binding site. The amplicon was digested with *Xba*I and cloned into the pRRK plasmid, which contains an *aphA3* cassette in the opposite orientation to the ribosomal genes, to obtain plasmid construct pRRK*arsB*. The direction of the insertion was confirmed by primers 16sarsB-F and 16sarsB-R. The construct with *arsB* in the same transcriptional direction as the ribosomal genes was selected and used as the suicide vector to insert the *arsB* gene into the chromosome of the *arsB* mutant. The complemented strains were selected on MH agar containing 30 µg/ml of kanamycin and were confirmed by PCR using primers 16sarsB-F and 16sarsB-R.

### Overexpression of *arsB* in *C. jejuni* NCTC 11168

The suicide plasmid pRRK*arsB* constructed for complementation was electroporated into wild-type *C. jejuni* NCTC 11168 wild type strain, resulting in the insertion of an extra copy of *arsB* in the chromosome. Transformants were selected on MH agar plates containing 30 µg/ml of kanamycin and confirmed by PCR using primers 16sarsB-F and 16sarsB-R.

### Real-time qRT-PCR

To determine if the *arsB* gene is inducible by arsenic compounds, *C. jejuni* NCTC 11168 was cultured in MH broth with or without added arsenite and arsenate for 20 h. The final concentrations of arsenite and arsenate in the culture were 0.125, 0.25, and 0.5 times of their corresponding MIC in NCTC 11168. Total RNA was extracted from three biological replicate cultures using the RNeasy mini kit (Qiagen) according to the protocol supplied with the product and further treated with the Turbo DNA-free kit (Ambion) to eliminate DNA contamination in each preparation. For real-time quantitative reverse transcription-PCR (qRT-PCR), primers arsB-F1 and arsB-R1 (Table 2) specific for *arsB* were designed using the Primer3 online interface (http://frodo.wi.mit.edu/). Real-time qRT-PCR analyses were conducted using the iScript one-step RT-PCR kit with SYBR green (Bio-Rad) along with the MyiQ iCycler real-time PCR detection system (Bio-Rad, Hercules, CA), and the16S rRNA gene was used for normalization as described in a previous publication [37]. Briefly, for each RNA template, to generate the standard curve for quantification of the target transcript, a 10-fold dilution series between 25 ng/µl and 0.0025 ng/µl were made and used for RT-PCR. Triplicate reactions in a volume of 15 µl were performed for each dilution of the RNA template. Thermal cycling conditions were as follows: 10 min at 50°C, 5 min at 60°C followed by 5 min at 95°C, and then 40 cycles of 10 s at 95°C and 30 s at 58°C. Melt-curve analysis was performed immediately after the amplification. Each specific amplicon was verified both by the presence of a single melting temperature peak and by the presence of a single band of expected size on agarose gel after electrophoresis. Cycle threshold values were determined with the MyiQ software (BioRad). The relative changes (n-fold) of transcription in *arsB* between the induced and noninduced samples were calculated using the 2^−ΔΔCT^ method as described by Livak and Schmittgen [38].

### Analysis of *ars* Gene Distribution by PCR

To determine the distribution of the *arsB* and *acr3* genes in various *C. jejuni* isolates, *arsB*-specific primers (arsB56F and arsB1185R) and *acr3*-specific primers (cje1733F and cje1733R) [6] were designed from the genomic sequence of *C. jejuni* NCTC 11168 and RM1221, respectively, and used in PCR analyses with the genomic templates of different *C. jejuni* strains and the Ex Taq polymerase (TaKaRa Bio Inc., Japan). These *C. jejuni* isolates were derived from human, chicken, and turkey.

## Results

### Genetic Features of *arsB*, *arsC2*, and *arsR3*

**Figure 1:**
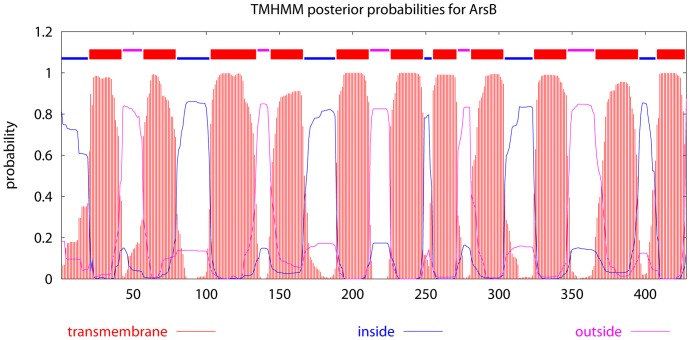
The membrane topologies of ArsB predicted by TMHMM. The transmembrane domains are shaded in red. The blue line indicates loops facing inside (cytoplasma), while the pink line depicts loops facing outside (periplasmic space). The numbers at the bottom indicate the amino acid numbers in ArsB.

**Figure 2:**
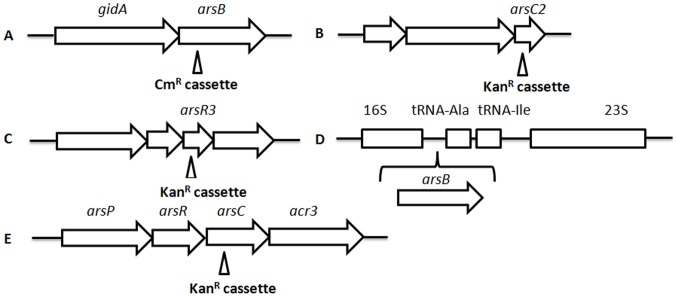
Diagrams showing the genomic localizations and mutant generation of various *ars* genes. (A) Genomic organization of *arsB* and inactivation of *arsB* by insertion of a choramphenicol resistance cassette. (B) Genomic localization of *arsC2* and inactivation of this gene by insertion of a kanamycin resistance cassette. (C) *arsR3* and its flanking gene. Inactivation of *arsR3* was accomplished by insertion of a kanamycin resistance cassette. (D) Complementation of the *arsB* mutant by insertion of an extra copy of the *arsB* gene downstream of 16S rRNA. (E) The *ars* operon identified in *C. jejuni* CB5-28 and inactivation of *arsC* by insertion of a kanamycin resistance cassette.

The *arsB* gene encodes a putative arsenic efflux membrane protein (428 amino acids) and shows amino acid sequence homology to ArsB in *Shewanella* sp. ANA-3 (32% identity; E = 8e−55) [39], *Staphylococcus aureus* (33% identity; E = 2e−65) [40], *Escherichia coli* (32% identity; E = 3e−54) [41]–[43], and *Acidithiobacillus caldus* (33% identity; E = 1e−57) [44]. ArsB contains eleven probable transmembrane helices predicted by TMHMM2.0 (Fig. 1). Analysis of several published genome sequences of *C. jejuni* strains showed that the *arsB* gene is conserved and immediately downstream of the *gidA* gene (Fig. 2A), which encodes a putative tRNA uridine 5-carboxymethylaminomethyl modification enzyme [45]. RT-PCR (using primers arsBgidAF and arsBgidAR) amplified a transcript spanning both *gidA* and *arsB*, suggesting that these two genes form an operon and are co-transcripted. *cj0717* encodes a small protein (109 aa), which is predicted to belong to the arsenate reductase (ArsC) family and the Yffb subfamily. Yffb is an uncharacterized bacterial protein encoded by the *yffb* gene, marginally similar to the amino-acid sequences of classical arsenate reductases (ArsC) (Fig. 2B). *cj0258* encodes an ORF of 81 aa, which is predicted to contain a helix-turn-helix motif at aa 35–56 and belongs to the *arsR* family [33], [45]. To differentiate *cj0258* from the *asrR* genes and *cj0717* from the *arsC* gene previously identified in *C. jejuni* [6], we named them as *arsR3* and *arsC2* in this study, respectively (Fig. 2B and C).

### Role of *arsB*, *arsC2,* and *arsR3* in Arsenic Resistance

To define the role of *arsB*, *arsC2*, and *arsR3* in arsenic resistance in *Campylobacter*, their insertional mutants were compared with the wild-type strain NCTC 11168 for susceptibility to arsenic compounds. According to the MIC results from the agar dilution method, inactivation of *arsB* resulted in 8- and 4-fold reduction in the MICs of arsenite and arsenate, respectively, while mutation of *arsC2* or *arsR3* did not affect the MICs of arsenite and arsenate (Table 3). All three mutants showed no changes in the MIC of roxarsone. Chromosomal complementation of *arsB* restored the MIC of arsenite to wild type, and over-expression of *arsB* showed 16-fold increase in the MIC of arsenite compared to the wild-type strain. Interestingly, chromosomal complementation could not restore the MIC of arsenate to the wild-type level and over expression of *arsB* showed no change in the MIC of arsenate compared to the wild-type strain. Furthermore, we transferred the *arsB* mutation to two additional *Campylobacter* strains (ATCC 33560 and CB5-28) by natural transformation. Inactivation of *arsB* in ATCC 33560 resulted in 8-fold reduction in the MICs of arsenite and had no affect on the MIC of arsenate and roxarsone (Table 3). Inactivation of *arsB* in CB5-28, which harbors the 4-gene *ars* operon as described in a previous study [6], did not affect the MICs of arsenite, arsenate, and roxarsone, suggesting the function of *arsB* in CB5-28 is masked by the fully functional *ars* operon.

**Table 3:** The MICs of roxarsone, arsenite and arsenate in various *C. jejuni* strains as determined by the agar dilution method*.

		MIC (µg/ml)	
Strains	Arsenite	Arsenate	Roxarsone
NCTC 11168	8	512	8
11168Δ*arsB*	1(↓8)	128(↓4)	8
11168Δ*arsC2*	8	512	8
11168Δ*arsR3*	8	512	8
11168Δ*arsB*Δ*arsC2*	1(↓8)	128(↓4)	8
11168Δ*arsB+arsB*	128	128	8
11168+*arsB*	128(↑16)	512	8
ATCC 33560	8	32	8
33560Δ*arsB*	1(↓8)	32	8
CB5-28	64	1024	64
CB5-28Δ*arsB*	64	1024	64
CB5-28Δ*arsC*	8	64	64
CB5-28Δ*arsB*Δ*arsC*	4(↓2)	64	64

* the numbers in parentheses indicate fold-changes, either increase (↑) or decrease (↓).

### Mutation of the *arsB* did not Affect the Susceptibility to the Other Antibiotics

To examine if *arsB*, *arsC2*, and *arsR3* are associated with resistance to other heavy metals and antibiotics, we compared the susceptibilities of the *arsB*, *arsC2*, and *arsR3* mutants with the wild-type strain to antimonate, copper sulfate, mercury bichloride, erythromycin, tetracycline, ethidium bromide, azithromycin, ciprofloxacin, florfenicol, telithromycin, and clindamycin using the broth microdilution method. The results showed no differences between the wild type and mutants in the susceptibilities to these compounds (data not shown), indicating that these genes do not confer resistance to other heavy metals and antibiotics.

### The *arsB* is Inducible by Arsenite and Arsenate

**Figure 3:**
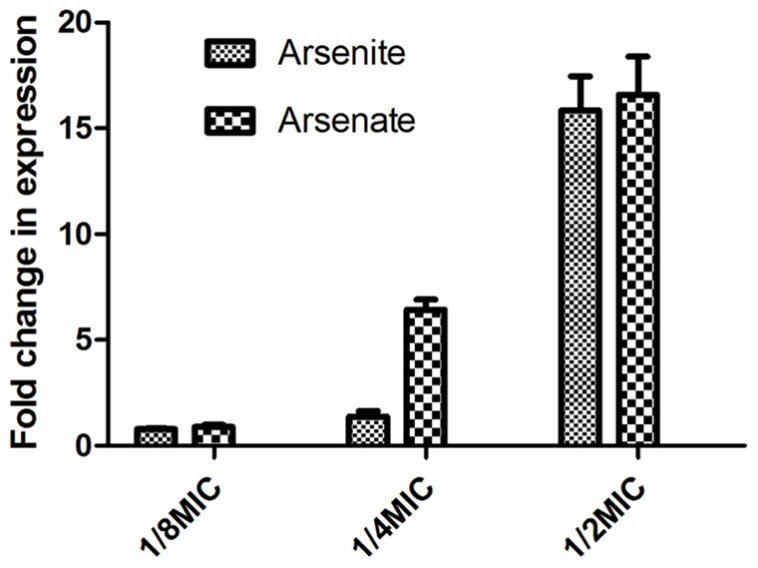
Dose-dependent induction of *arsB* in 11168 by arsenite and arsenate. The concentrations of the arsenic compounds supplemented into the culture media are labeled at the bottom of the panel.

To determine if the expression of the *arsB* is inducible by arsenic compounds, strain NCTC11168 was cultured in MH broth with different concentrations of arsenite and arsenate. The transcription levels of *arsB* in these cultures were compared with those grown in MH broth without arsenic compounds using real time qRT-PCR. As shown in Figure 3, the expression of *arsB* was induced in a dose-dependent manner. At 0.5 times of MIC, both arsenite and arsenate produced approximately 16-fold induction in the expression of *arsB*. This result clearly indicates that the *arsB* gene in *Campylobacter* is inducible by both arsenite and arsenate.

### Distribution of *arsB* and *acr3* Genes in *Campylobacter* Isolates

Data described above indicated that ArsB contributes to arsenic resistance in *C. jejuni*. Additionally, Acr3 is associated with high-level of arsenic resistance in certain *Campylobacter* strains [6]. We determined the distribution of the *arsB* and *acr3* genes in various *Campylobacter* isolates of different animal origins. As shown in Table 4, *arsB* was present in 76 of the 98 isolates examined in this study, while *acr3* were present in 58 of the 98 isolates. Interestingly, all the tested strain contains at least one of the two genes. Furthermore, *arsB* is more prevalent in the chicken (97.1%) and human (92.0%) isolates than in the turkey isolates (50.0%) (*p*<0.0001 and *p*<0.005), while the prevalence of *acr3* is higher in the turkey isolates (84.2%) than in the chicken (45.7%) (*p*<0.005) and human (40.0%) (*p*<0.005) isolates.

**Table 4:** Distribution of *arsB* and *acr3* in *C. jejuni* isolates of different origins.

Source of isolates	Total number	*arsB*-positive	*acr3*-positive	Positive with both*arsB* and *acr3*	Positive with either*arsB* or *acr3*
Chicken	35	34 (97.1%)	16 (45.7%)	15 (42.9%)	35 (100.0%)
Turkey	38	19 (50.0%)	32 (84.2%)	13 (34.2%)	38 (100.0%)
Human	25	23 (92.0%)	10 (40.0%)	8 (32.0%)	25 (100.0%)
total	98	76 (77.6%)	58 (59.2%)	36 (36.7%)	98 (100.0%)

## Discussion

The results from this study identified ArsB involved in arsenic resistance in *C. jejuni.* This conclusion is based on the following findings: first, inactivation of *arsB* resulted in reduced resistance to both arsenite and arsenate; second, complementation of the *arsB* mutant restored the MIC of arsenite (but not arsenate) to that of the wild-type strain; and third, overexpression of *arsB* in *C. jejuni* 11168 increased the MIC of arsenite by 16-fold, but did not affect the MIC of arsenate. These results suggest that ArsB in *C. jejuni* contributes resistance to arsenite, but not for arsenate. However, arsenate can be converted to arsenite by ArsC in bacteria including *C. jejuni,* where arsenite can be subsequently extruded by ArsB and Acr3 [6]. Thus, ArsB contributes to the resistance to arsenate in an indirect manner. These results are consistent with the *arsB* findings in other bacterial species.

The ArsB in *C. jejuni* shares homology with the other members of the ArsB family. ArsB is employed by many bacteria as an arsenic detoxification method and is proposed to have 12 membrane-spanning regions [46]. ArsB appears to be an uniporter which extrudes As(III) at a moderate rate using membrane potential. In some cases, with the help from ArsA (ATPase), ArsB can extrude As(III) more efficiently [5]. Several previous studies also showed that Sb(III) is a substrate for certain ArsB transpoters [47]. In this study, we found that the ArsB in *C. jejuni* does not play a role in the resistance to other heavy metals and antibiotics. The inability of *C. jejuni* ArsB to extrude Sb(III) is different from the result reported in other bacteria [47] and suggests that the ArsB in *C. jejuni* is more or less unique. Indeed, the predicted transmembrane topology of the ArsB in *C. jejuni* contains 11 transmembrane domains, instead of 12 of typical ArsB proteins, which might explain the difference in substrate specificities.

The contributions of *arsB* to arsenic resistance vary in different *Campylobacter* strains. The role of ArsB in mediating arsenic resistance to As(III) is more prominent in those strains that lack the *ars* operon, such as NCTC 11168 and ATCC 33560 (Table 3). On the contrary, inactivation of *arsB* in the highly resistant CB5-28 strain (containing an *ars* operon) did not change the MIC of arsenite (Table 3). To test if the function of ArsB is masked by the presence of the *ars* operon, we constructed an *arsB* and *arsC* double knockout strain (CB5-28Δ*arsB*Δ*ars*C) in the CB5-28Δ*arsC* background [6]. Compared to CB5-28Δ*arsC*, CB5-28Δ*arsB*Δ*ars*C showed 2-fold reduction in the MIC of arsenite, but not 8-fold reduction as observed in NCTC 11168 (Table3). This could be explained by the fact that the polar effect caused by the *arsC* mutation did not totally inactivate the function of *acr3* and residual expression of *acr3* still existed in the *arsC* mutant compared with that in the wild-type strain [6]. Thus, the residual expression of *acr3* could still play a role in arsenite resistance. These results suggest that the function of *arsB* is most likely masked in those *C. jejuni* strains harboring a fully functional *ars* operon.

The level of arsenic resistance mediated by ArsB in *C. jejuni* is not as high as that mediated by the *ars* operon. This could be explained for two reasons. The published data in other bacteria indicated that ArsB functions more efficiently when facilitated by ArsA (ATPase) [5], [16]. However, analysis of the whole genomes of *C. jejuni* did not identify an *arsA* homology in the organism. Thus, the lack of *arsA* in *C. jejuni* might reduce the efflux ability of ArsB. In addition, the expression level of *arsB* might be another factor affecting its contribution to arsenic resistance. As show in Table 3, artificial overexpression of *arsB* in *C. jejuni* NCTC 11168 resulted in a drastic increase in the resistance to arsenite, to a level that is even higher than the resistance conferred by the *ars* operon. These findings suggest that ArsB mediated arsenic resistance level in *Campylobacter* is mainly dependent on the expression level of *arsB*.

The putative *arsR (arsR3)* gene did not contribute to arsenic resistance in *C. jejuni* NCTC 11168. As a transcriptional repressor, ArsR modulates the expression of *ars* genes through interaction with the arsenite substrate [6]. In this study, the induction experiment revealed that addition of arsenite or arsenate in culture media induced the expression of *arsB*, and the induction was dose-dependent (Fig. 3). Thus, we speculated that the expression of *arsB* is modulated by an ArsR like regulator. However, inactivation of *arsR3*, which is separated from the *arsB* gene on chromosome, did not affect the expression of *arsB* in *C. jejuni* NCTC 11168, suggesting that the expression of *arsB* is not modulated by *arsR3* and is likely regulated by an unknown mechanism.

The putative *arsC (arsC2)* gene did not contribute to arsenic resistance *C. jejuni* NCTC 11168. Conversion of AS(V) to AS(III) by arsenate reductase and then extrusion by arsenite transporters is an important detoxification mechanism used by many bacterial organisms [6], [15]. The previously characterized *ars* operon in *C. jejuni* contains an *arsC*, which mediates arsenic resistance in *Campylobacter* [6]. Inactivation of *arsC2* in *C. jejuni* NCTC 11168 did not change the susceptibility to arsenic compounds. Additionally, we inactivated *arsC2* in the *arsB* mutant background of *C. jejuni* NCTC 11168, and the the *arsB* and *arsC* double knockout did not further alter the resistance to arsenate compared to the *arsB* mutant (data not shown), further suggesting that *arsC2* is not involved in arsenic resistance in *Campylobacter*.

As mentioned in the introduction, organic arsenic compounds (roxarsone and p-arsanilic acid) are extensively used as feed additives in the poultry industry and *Campylobacter* is exposed to the selection pressure. ArsB is a putative efflux transporter for inorganic arsenic and does not seem to directly contribute to the resistance to roxarsone (Table 3). However, roxarsone is converted into inorganic species such as AS(V) and AS(III) in poultry litter [32]. Thus, ArsB is expected to facilitate *Campylobacter* adaptation to the toxic effect of roxarsone in an indirect manner. To date, the identified mechanisms of arsenic resistance in bacteria are all related to detoxification of inorganic arsenic, and the efflux transporters that directly extrude organic arsenic compounds have not been reported.

Interestingly, the distribution of both *arsB* and *acr3* in human isolates is similar to those in chicken isolates, but differ from those in turkey isolates (Table 4). According to a report from American Meat Institute on April 2009, per capita consumption of chicken was five times more than that of turkey in 2007. In addition, poultry is the main reservoir for human *C. jejuni* infections. Thus, the big portion of *Campylobacter* infections is probably caused by consumption of chicken. This might explain that the presence of *ars* genes in human isolates is similar to those in chicken isolates. Furthermore, the results revealed a broad distribution of *arsB* and *acr3* genes (*ars* operon) in *C. jejuni* isolates of different animal origins (Table 4) and suggest that at least one of the two genes is required for the adaptation of *Campylobacter* in arsenic-rich niches. These findings provide new insights into the adaptive mechanisms of *Campylobacter* in the poultry production system.

## References

[1] Basu A, Mahata J, Gupta S, Giri AK (2001) Genetic toxicology of a paradoxical human carcinogen, arsenic: a review. Mutat Res 488: 171–194. 11344043 10.1016/s1383-5742(01)00056-4

[2] Oremland RS, Stolz JF (2003) The ecology of arsenic. Science 300: 939–944. 12738852 10.1126/science.1081903

[3] Flora SJ (2011) Arsenic-induced oxidative stress and its reversibility. Free Radic Biol Med 51: 257–281. 21554949 10.1016/j.freeradbiomed.2011.04.008

[4] Yang HC, Cheng J, Finan TM, Rosen BP, Bhattacharjee H (2005) Novel pathway for arsenic detoxification in the legume symbiont *Sinorhizobium meliloti* . J Bacteriol 187: 6991–6997. 16199569 10.1128/JB.187.20.6991-6997.2005 PMC1251620

[5] Rosen BP (2002) Biochemistry of arsenic detoxification. FEBS Lett 529: 86–92. 12354618 10.1016/s0014-5793(02)03186-1

[6] Wang L, Jeon B, Sahin O, Zhang Q (2009) Identification of an arsenic resistance and arsenic-sensing system in *Campylobacter jejuni* . Appl Environ Microbiol 75: 5064–5073. 19502436 10.1128/AEM.00149-09 PMC2725487

[7] Qin J, Rosen BP, Zhang Y, Wang G, Franke S, et al (2006) Arsenic detoxification and evolution of trimethylarsine gas by a microbial arsenite S-adenosylmethionine methyltransferase. Proc Natl Acad Sci U S A 103: 2075–2080. 16452170 10.1073/pnas.0506836103 PMC1413689

[8] Butcher BG, Deane SM, Rawlings DE (2000) The chromosomal arsenic resistance genes of *Thiobacillus ferrooxidans* have an unusual arrangement and confer increased arsenic and antimony resistance to *Escherichia coli* . Appl Environ Microbiol 66: 1826–1833. 10788346 10.1128/aem.66.5.1826-1833.2000 PMC101419

[9] Butcher BG, Rawlings DE (2002) The divergent chromosomal ars operon of *Acidithiobacillus ferrooxidans* is regulated by an atypical ArsR protein. Microbiology 148: 3983–3992. 12480902 10.1099/00221287-148-12-3983

[10] Cai J, Salmon K, DuBow MS (1998) A chromosomal ars operon homologue of *Pseudomonas aeruginosa* confers increased resistance to arsenic and antimony in *Escherichia coli* . Microbiology 144 (Pt 10): 2705–2713. 10.1099/00221287-144-10-2705 9802012

[11] Diorio C, Cai J, Marmor J, Shinder R, DuBow MS (1995) An *Escherichia coli* chromosomal ars operon homolog is functional in arsenic detoxification and is conserved in gram-negative bacteria. J Bacteriol 177: 2050–2056. 7721697 10.1128/jb.177.8.2050-2056.1995 PMC176848

[12] Lopez-Maury L, Florencio FJ, Reyes JC (2003) Arsenic sensing and resistance system in the cyanobacterium *Synechocystis* sp. strain PCC 6803. J Bacteriol 185: 5363–5371. 12949088 10.1128/JB.185.18.5363-5371.2003 PMC193754

[13] Sato T, Kobayashi Y (1998) The ars operon in the skin element of *Bacillus subtilis* confers resistance to arsenate and arsenite. J Bacteriol 180: 1655–1661. 9537360 10.1128/jb.180.7.1655-1661.1998 PMC107075

[14] Suzuki K, Wakao N, Kimura T, Sakka K, Ohmiya K (1998) Expression and regulation of the arsenic resistance operon of *Acidiphilium multivorum* AIU 301 plasmid pKW301 in *Escherichia coli* . Appl Environ Microbiol 64: 411–418. 9464374 10.1128/aem.64.2.411-418.1998 PMC106059

[15] Ji G, Silver S (1992) Reduction of arsenate to arsenite by the ArsC protein of the arsenic resistance operon of *Staphylococcus aureus* plasmid pI258. Proc Natl Acad Sci U S A 89: 9474–9478. 1409657 10.1073/pnas.89.20.9474 PMC50154

[16] Dey S, Rosen BP (1995) Dual mode of energy coupling by the oxyanion-translocating ArsB protein. J Bacteriol 177: 385–389. 7814328 10.1128/jb.177.2.385-389.1995 PMC176602

[17] Saltikov CW, Olson BH (2002) Homology of Escherichia coli R773 *arsA*, *arsB*, and *arsC* genes in arsenic-resistant bacteria isolated from raw sewage and arsenic-enriched creek waters. Appl Environ Microbiol 68: 280–288. 11772637 10.1128/AEM.68.1.280-288.2002 PMC126541

[18] Rosenstein R, Nikoleit K, Gotz F (1994) Binding of ArsR, the repressor of the *Staphylococcus xylosus* (pSX267) arsenic resistance operon to a sequence with dyad symmetry within the ars promoter. Mol Gen Genet 242: 566–572. 8121414 10.1007/BF00285280

[19] Wu J, Rosen BP (1991) The ArsR protein is a trans-acting regulatory protein. Mol Microbiol 5: 1331–1336. 1838573 10.1111/j.1365-2958.1991.tb00779.x

[20] Zhang YB, Monchy S, Greenberg B, Mergeay M, Gang O, et al (2009) ArsR arsenic-resistance regulatory protein from *Cupriavidus metallidurans* CH34. Antonie Van Leeuwenhoek 96: 161–170. 19238575 10.1007/s10482-009-9313-z

[21] Murphy JN, Saltikov CW (2009) The ArsR repressor mediates arsenite-dependent regulation of arsenate respiration and detoxification operons of *Shewanella* sp. strain ANA-3. J Bacteriol 191: 6722–6731. 19717602 10.1128/JB.00801-09 PMC2795299

[22] Ajees AA, Yang J, Rosen BP (2011) The ArsD As(III) metallochaperone. Biometals 24: 391–399. 21188475 10.1007/s10534-010-9398-x PMC3773603

[23] Yang J, Rawat S, Stemmler TL, Rosen BP (2010) Arsenic binding and transfer by the ArsD As(III) metallochaperone. Biochemistry 49: 3658–3666. 20361763 10.1021/bi100026a PMC2920133

[24] Lin YF, Walmsley AR, Rosen BP (2006) An arsenic metallochaperone for an arsenic detoxification pump. Proc Natl Acad Sci U S A 103: 15617–15622. 17030823 10.1073/pnas.0603974103 PMC1622871

[25] Neyt C, Iriarte M, Thi VH, Cornelis GR (1997) Virulence and arsenic resistance in *Yersiniae* . J Bacteriol 179: 612–619. 9006011 10.1128/jb.179.3.612-619.1997 PMC178738

[26] Ye J, Yang HC, Rosen BP, Bhattacharjee H (2007) Crystal structure of the flavoprotein ArsH from *Sinorhizobium meliloti* . FEBS Lett 581: 3996–4000. 17673204 10.1016/j.febslet.2007.07.039 PMC1989112

[27] Scallan E, Hoekstra RM, Angulo FJ, Tauxe RV, Widdowson MA, et al (2011) Foodborne illness acquired in the United States–major pathogens. Emerg Infect Dis 17: 7–15. 21192848 10.3201/eid1701.P11101 PMC3375761

[28] Ruiz-Palacios GM (2007) The health burden of *Campylobacter* infection and the impact of antimicrobial resistance: playing chicken. Clin Infect Dis 44: 701–703. 17278063 10.1086/509936

[29] Humphrey T, O'Brien S, Madsen M (2007) *Campylobacters* as zoonotic pathogens: a food production perspective. Int J Food Microbiol 117: 237–257. 17368847 10.1016/j.ijfoodmicro.2007.01.006

[30] Chapman HD, Johnson ZB (2002) Use of antibiotics and roxarsone in broiler chickens in the USA: analysis for the years 1995 to 2000. Poult Sci 81: 356–364. 11902412 10.1093/ps/81.3.356

[31] Garbarino JR, Bednar AJ, Rutherford DW, Beyer RS, Wershaw RL (2003) Environmental fate of roxarsone in poultry litter. I. Degradation of roxarsone during composting. Environ Sci Technol 37: 1509–1514. 12731831 10.1021/es026219q

[32] Jackson BP, Bertsch PM, Cabrera ML, Camberato JJ, Seaman JC, et al (2003) Trace element speciation in poultry litter. J Environ Qual 32: 535–540. 12708677 10.2134/jeq2003.5350

[33] Parkhill J, Wren BW, Mungall K, Ketley JM, Churcher C, et al (2000) The genome sequence of the food-borne pathogen *Campylobacter jejuni* reveals hypervariable sequences. Nature 403: 665–668. 10688204 10.1038/35001088

[34] (2006) Clinical and Laboratory Standards Institute. Performance standards for antimicrobial susceptibility testing; 16th informational supplement. CLSI M100-S16. Clinical and Laboratory Standards Institute, Wayne, PA.

[35] Lin J, Michel LO, Zhang Q (2002) CmeABC functions as a multidrug efflux system in *Campylobacter jejuni* . Antimicrob Agents Chemother 46: 2124–2131. 12069964 10.1128/AAC.46.7.2124-2131.2002 PMC127319

[36] Muraoka WT, Zhang Q (2011) Phenotypic and Genotypic Evidence for L-Fucose Utilization by *Campylobacter jejuni* . J Bacteriol 193: 1065–1075. 21193610 10.1128/JB.01252-10 PMC3067607

[37] Lin J, Cagliero C, Guo B, Barton YW, Maurel MC, et al (2005) Bile salts modulate expression of the CmeABC multidrug efflux pump in *Campylobacter jejuni* . J Bacteriol 187: 7417–7424. 16237025 10.1128/JB.187.21.7417-7424.2005 PMC1272998

[38] Livak KJ, Schmittgen TD (2001) Analysis of relative gene expression data using real-time quantitative PCR and the 2(-Delta Delta C(T)) Method. Methods 25: 402–408. 11846609 10.1006/meth.2001.1262

[39] Saltikov CW, Cifuentes A, Venkateswaran K, Newman DK (2003) The ars detoxification system is advantageous but not required for As(V) respiration by the genetically tractable *Shewanella* species strain ANA-3. Appl Environ Microbiol 69: 2800–2809. 12732551 10.1128/AEM.69.5.2800-2809.2003 PMC154534

[40] Ji G, Silver S (1992) Regulation and expression of the arsenic resistance operon from *Staphylococcus aureus* plasmid pI258. J Bacteriol 174: 3684–3694. 1534328 10.1128/jb.174.11.3684-3694.1992 PMC206058

[41] Chen CM, Misra TK, Silver S, Rosen BP (1986) Nucleotide sequence of the structural genes for an anion pump. The plasmid-encoded arsenical resistance operon. J Biol Chem 261: 15030–15038. 3021763

[42] San Francisco MJ, Tisa LS, Rosen BP (1989) Identification of the membrane component of the anion pump encoded by the arsenical resistance operon of R-factor R773. Mol Microbiol 3: 15–21. 2523997 10.1111/j.1365-2958.1989.tb00098.x

[43] Tisa LS, Rosen BP (1990) Molecular characterization of an anion pump. The ArsB protein is the membrane anchor for the ArsA protein. J Biol Chem 265: 190–194. 1688427

[44] Tuffin IM, de Groot P, Deane SM, Rawlings DE (2005) An unusual Tn21-like transposon containing an ars operon is present in highly arsenic-resistant strains of the biomining bacterium *Acidithiobacillus caldus* . Microbiology 151: 3027–3039. 16151213 10.1099/mic.0.28131-0

[45] Gundogdu O, Bentley SD, Holden MT, Parkhill J, Dorrell N, et al (2007) Re-annotation and re-analysis of the *Campylobacter jejuni* NCTC11168 genome sequence. BMC Genomics 8: 162. 17565669 10.1186/1471-2164-8-162 PMC1899501

[46] Wu J, Tisa LS, Rosen BP (1992) Membrane topology of the ArsB protein, the membrane subunit of an anion-translocating ATPase. J Biol Chem 267: 12570–12576. 1535622

[47] Rosen BP (1999) Families of arsenic transporters. Trends Microbiol 7: 207–212. 10354596 10.1016/s0966-842x(99)01494-8

[48] Wang Y, Taylor DE (1990) Chloramphenicol resistance in *Campylobacter coli*: nucleotide sequence, expression, and cloning vector construction. Gene 94: 23–28. 2227449 10.1016/0378-1119(90)90463-2

[49] Yanisch-Perron C, Vieira J, Messing J (1985) Improved M13 phage cloning vectors and host strains: nucleotide sequences of the M13mp18 and pUC19 vectors. Gene 33: 103–119. 2985470 10.1016/0378-1119(85)90120-9

[50] Wosten MM, Boeve M, Koot MG, van Nuenen AC, van der Zeijst BA (1998) Identification of *Campylobacter jejuni* promoter sequences. J Bacteriol 180: 594–599. 9457862 10.1128/jb.180.3.594-599.1998 PMC106926

